# Metabolic Stroke: Atypical Presentation of Succinic Semialdehyde Dehydrogenase Deficiency

**DOI:** 10.1002/jmd2.70071

**Published:** 2026-02-01

**Authors:** Sharmila Kiss, Richard J. Leventer, Cormac Duff, Emma Macdonald‐Laurs, Olivia‐Paris Quinn, Fahaz Nazer, Michelle Cao, Abisha Srikumar, Jenzen Dalina, Mary Eggington, Joy Yaplito‐ Lee

**Affiliations:** ^1^ Department of Metabolic Medicine The Royal Children's Hospital Parkville Victoria Australia; ^2^ Department of Neurology The Royal Children's Hospital Parkville Victoria Australia; ^3^ Victorian Clinical Genetics Services, Murdoch Children's Research Institute Parkville Victoria Australia; ^4^ Department of Paediatrics University of Melbourne Melbourne Victoria Australia; ^5^ Neuroscience Stream, Murdoch Children's Research Institute Parkville Victoria Australia

## Abstract

Succinic semialdehyde dehydrogenase (SSADH) deficiency is a rare autosomal recessive neurometabolic disorder caused by biallelic pathogenic variants in *ALDH5A1*, encoding the mitochondrial enzyme SSADH. This enzyme catalyses the conversion of succinic semialdehyde to succinic acid in the γ‐aminobutyric acid (GABA) degradation pathway. SSADH deficiency leads to the accumulation of neurotoxic metabolites, including γ‐hydroxybutyrate (GHB), and presents with developmental delay, hypotonia, ataxia, seizures, behavioral disturbances, and intellectual disability. We report a 10‐month‐old Caucasian male with global developmental delay, central hypotonia, and delayed motor milestones. He presented acutely with left‐sided hemiplegia following irritability and vomiting. Brain MRI showed bilateral (right > left) T2 hyperintensities and diffusion restriction in the globus pallidus. Urine organic acid analysis via gas chromatography–mass spectrometry revealed markedly elevated 4‐hydroxybutyric acid and 4,5‐dihydroxyhexanoic lactone, pathognomonic for SSADH deficiency. Molecular testing identified compound heterozygous *ALDH5A1* variants: c.278G>T p.(Cys93Phe) and c.612G>A p.(Trp204*), both previously reported as pathogenic. Parental segregation confirmed trans configuration. Three weeks postillness, he developed focal seizures, which have remained well controlled on levetiracetam. His seizure onset in infancy is notably earlier than the typical early childhood onset (~9 years) reported in SSADH deficiency. This case expands the phenotypic spectrum of SSADH deficiency to include metabolic stroke as a presenting feature in infancy and highlights the importance of early recognition and molecular confirmation to guide management and emerging therapeutic strategies.

## Key Points

- This case expands the phenotypic spectrum of SSADH deficiency by identifying metabolic stroke (presenting as acute hemiplegia) as a potential initial manifestation in infancy.
- The report highlights an unusually early onset of focal seizures at 10 months of age, significantly younger than the reported median age of seizure onset (approximately 9 years) in this disorder.

## 1 Introduction

Succinic semialdehyde dehydrogenase (SSADH) deficiency is a rare autosomal recessive metabolic disorder caused by biallelic pathogenic variants in the *ALDH5A1* gene, which encodes the mitochondrial enzyme SSADH [1]. This enzyme plays a critical role in degrading the inhibitory neurotransmitter γ‐aminobutyric acid (GABA), catalyzing the conversion of succinic semialdehyde (SSA) to succinic acid. SSADH deficiency leads to the accumulation of SSA and its reduction product, γ‐hydroxybutyrate (GHB), in the central nervous system and other tissues [2].

The global prevalence is estimated between 1 in 223 000 and 1 in 564 000 individuals [3]. Fewer than 500 cases have been reported, though the true incidence is likely underestimated due to diagnostic challenges and phenotypic variability.

Clinical features of SSADH deficiency are nonspecific and highly heterogenous, including developmental delay, hypotonia, cognitive and adaptive disabilities, communication and language deficits, epilepsy, autism spectrum disorder, movement disorders (such as ataxia, dystonia, and exertional dyskinesia), sleep disturbances, attention problems, anxiety, and obsessive‐compulsive traits [4]. Severity can vary from mild impairment to a progressive neurodegenerative condition with drug‐resistant epilepsy [5]. Biochemical investigations often reveal elevated levels of GHB in urine, a hallmark finding detected via gas chromatography–mass spectrometry (GC–MS). In some cases, plasma and cerebrospinal fluid (CSF) may also show increased GHB concentrations. Routine blood tests are typically unremarkable, but CSF studies may reveal increased GABA, reflecting impaired SSA catabolism [3, 6, 7].

Despite increasing recognition, the pathophysiological mechanisms remain incompletely understood, with poor genotype–phenotype correlation, and treatment options are currently limited to symptomatic management. Advances in genetic diagnostics and experimental therapeutics including gene therapy, enzyme replacement strategies, and GABAergic modulators have renewed interest in elucidating the molecular and cellular consequences of SSADH deficiency.

Magnetic resonance imaging (MRI) is useful for identifying structural brain abnormalities and monitoring disease progression [8]. MRI often shows increased T2‐weighted signal intensity in the globus pallidus, cerebellar dentate nucleus, and subthalamic nucleus, often accompanied by varying degrees of cerebral and cerebellar atrophy [9, 10, 11]. Additionally, diffusion restriction and abnormal perfusion within the globus pallidus have been observed in isolated cases [8], though most findings stem from case reports, limiting the generalizability of these findings.

Electroencephalogram (EEG), by contrast, does not meaningfully aid diagnosis, as findings are typically nonspecific, such as generalized and focal epileptiform discharges, photosensitivity, and background slowing [12]. Unlike certain neurometabolic or genetic conditions (e.g., pyridoxine deficiency or Angelman syndrome), EEG features in SSADH do not clearly suggest a metabolic etiology.

## 2 Case Report

We present a 10‐month‐old male infant born to nonconsanguineous parents of European‐Australian ethnicity. He was born at term with normal growth parameters following an unremarkable pregnancy and delivery. Morphology scans at 20 weeks showed a dilated coronary sinus raising concerns for a ventricular septal defect. Prenatal genetic testing was not performed. The patient was delivered at 38 + 4 weeks gestation via spontaneous vaginal delivery, following induction of labor due to the antenatal cardiac findings. Birth weight was 3.26 kg. No resuscitation was required. Postnatally, he was admitted to the Special Care Unit for further evaluation. Postnatal echocardiography demonstrated a common anatomical variant of a bilateral superior vena cava (SVC), with right SVC draining to right atrium, and left SVC to roofed coronary sinus. He remained stable without the need for respiratory or feeding support.

A solitary subcutaneous vascular lesion was noted on the left fronto‐parietal scalp. Renal ultrasound revealed a small renal cyst, which remained stable in size on follow‐up imaging. These are thought to be incidental findings. Ophthalmologic examination (performed after review in Neurology clinic) identified retinal hemorrhages, attributed to birth trauma, with no additional abnormalities detected.

At 4 months old he was referred to a physiotherapist with concerns regarding significant head lag that remains comparable to a newborn, right‐sided plagiocephaly, and hypotonia. When first assessed at the outpatient neurology clinic at 9 months, he was unable to hold up his head without support, roll over, or bear weight through his arms in a prone position. Although he could reach for objects, he was unable to grasp or bring them to his mouth. He could coo but had not yet begun babbling or shown signs of stranger anxiety. He had delayed feeding skills and was uninterested in solids, continuing to breastfeed exclusively. He slept more than is typical for a 9‐month‐old, napping every 1–3 h. Neurological examination demonstrated reduced ability to fix and follow, central and peripheral hypotonia with reduced but symmetric antigravity movements but preserved deep tendon reflexes. There were no cranial nerve abnormalities, no signs of hemiparesis, or evidence of a movement disorder. Growth parameters were within normal limits. Investigations for central hypotonia including ammonia, plasma amino acids, lactate, urine metabolic screen, microarray, *SMN1* copy number, DMPK triplet repeats, methylation studies, and MRI brain were requested. Prior to his acute presentation at age 9 months, he experienced 1 day of mild irritability, nasal congestion, and vomiting. There was no history of toxic ingestion. He presented to the local Emergency Department with an 8 h history of progressive left‐sided hemiparesis, initially affecting the left upper limb and subsequently involving the left lower limb. On examination, he demonstrated worsening central hypotonia and absence of spontaneous movement in the left upper limb. Power was reduced to 1/5 in the left upper limb and 4/5 in the left lower limb. Reflexes were symmetric and plantar responses equivocal.

**FIGURE 1:**
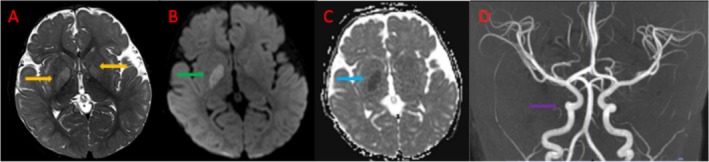
MRI and MRA Findings at age 9 months during the acute episode. (A) Axial T2‐weighted MRI showing T2‐signal hyperintensity of the globus pallidus bilaterally (right more than left) (orange arrows). (B) Axial diffusion weighted imaging (DWI) showing diffusion restriction of the right globus pallidus (green arrow). (C) Axial apparent diffusion coefficient (ADC) MRI showing a decrease in ADC value correlating with DWI changes (blue arrow). (D) MRA reconstruction showing relative tortuosity of the internal carotid arteries at the skull base (purple arrow).

Brain MRI demonstrated T2 hyperintensities in the globi pallidi bilaterally with mild parenchymal swelling and diffusion restriction of the right globus pallidus consistent with the left hemiparetic changes. (Figure 1) Time‐of‐flight angiogram demonstrated nonspecific relative tortuosity of the internal carotid arteries at the skull base but was otherwise unremarkable with patent vessels. (Figure 1) MRI spectroscopy was not performed. These findings were consistent with either bilateral ischaemic changes or a metabolic stroke. Prothrombotic workup and vessel wall imaging on MRI for focal cerebral arteriopathy were unremarkable. Urine metabolic screening by tandem MS and urine organic acids by GC–MS identified markedly elevated 4‐hydroxybutyric acid and 4,5 dihydroxyhexanoic lactone, pathognomonic for SSADH.

Given the specific diagnosis *t* suggested by urine organic acids, single gene testing was organized which identified two pathogenic variants in *ALDH5A1: ALDH5A1:* c.278G>T, p.(Cys93Phe), and c.612G>A, p.(Trp204*). Both these variants have been reported in several patients with SSADH in homozygous and compound heterozygous states. Segregation confirmed that each parent carried one of the variants.

The patient was discharged home with coordinated follow‐up involving Metabolic, Neurology, Genetics, and Ophthalmology services. His left‐sided weakness resolved after 5 days with supportive care. Three weeks after discharge, he developed focal seizures characterized by behavioral arrest with dystonic posturing of the right upper limb, associated with multifocal spike and polyspike–wave discharges on EEG. The seizures have remained well controlled with levetiracetam, without further evolution or developmental regression.

At follow‐up, he continued to demonstrate marked hypotonia and severe global developmental delay, consistent with his prestroke baseline. He was unable to sit unsupported at 15 months of age. Neurological examination remained symmetric, with no upper motor neuron signs. Follow‐up neuroimaging has not been performed.

He continues to receive multidisciplinary allied health support, including physiotherapy, occupational therapy, and speech pathology, both as an inpatient and outpatient. The family is not enrolled in any clinical trials or natural history studies.

## 3 Discussion

The presentation of a metabolic stroke in infancy, as seen in our case, represents a rare and atypical manifestation of SSADH deficiency. To date, only one prior report by Yoganathan et al. [13] describing a 15‐month‐old with acute‐onset right hemiparesis following a diarrhoeal illness, and MRI showed diffusion restriction in the posterior limb of the internal capsule, consistent with an acute metabolic stroke. The patient made a near‐complete recovery with supportive care [13].

Our patient presented earlier, at 10 months of age, with acute left hemiparesis during a febrile illness. The duration of symptoms exceeded 24 h, arguing against a transient ischaemic attack and supporting a metabolic stroke. He made a full neurological recovery, consistent with the reversible metabolic rather than structural nature. MRI showed symmetrical T2 hyperintensities in the globus pallidus without diffusion restriction or vascular abnormalities, favoring a metabolic rather than ischemic etiology. Symmetrical basal‐ganglia involvement is typical in SSADH deficiency. Unlike the Yoganathan case, our patient demonstrated persistent developmental delay, suggesting that a younger age of onset or basal‐ganglia involvement may be associated with poorer long‐term outcomes. Our case expands the recognized phenotypic spectrum of SSADH deficiency [1, 7, 8, 14, 15].

The patient's seizures were most likely secondary to the prior stroke, though subtle earlier episodes may have gone unrecognized. Given the mild seizure phenotype and reassuring EEG, further genetic investigation was not indicated but would be considered if seizures became frequent, drug‐resistant, or associated with regression.

While stroke is not considered characteristic of SSADH deficiency, it is a recognized complication of other inborn errors of metabolism (IEMs). Mitochondrial disorders such as MELAS (Mitochondrial Encephalomyopathy, Lactic Acidosis, and Stroke‐like episodes) often cause transient focal deficits linked to energy failure and impaired nitric oxide metabolism [13, 16]. Urea cycle disorders may trigger cerebral oedema and focal deficits during hyperammonemic crises. Similarly, striatal injury in glutaric aciduria type I, or metabolic crises in methylmalonic, propionic, isovaleric aciduria, and homocystinuria, can mimic stroke [13]. Our case adds SSADH deficiency to this group of IEMs in which acute focal neurological deficits can arise during illness or metabolic stress.

Current international guidelines (2024) do not list stroke as a recognized feature of SSADH deficiency [16]. SSADH deficiency is typically associated with global developmental delay, hypotonia, behavioral difficulties, seizures, and movement disorders. However, our case, together with the report by Yoganathan et al. suggests that metabolic stroke, while rare, should be considered in infants presenting with acute hemiparesis when conventional stroke workup is inconclusive.

The mechanisms underlying stroke‐like episodes in SSADH deficiency remain uncertain. Excess GABA and GHB due to impaired catabolism may exert neurotoxic effects through redox imbalance, oxidative stress, impaired autophagy, and osmotic dysregulation [8, 10, 11, 14].

Although SSADH deficiency usually causes diffuse encephalopathy, several features may predispose to focal injury. The basal ganglia, especially the globus pallidus, are highly metabolically active and enriched in GABAergic synapses, making them particularly vulnerable to accumulation of GABA/GHB. During febrile or catabolic stress, susceptibility is amplified by redox stress or impaired energy metabolism. Abnormal GABA/GHB levels may also disrupt neurovascular coupling, causing transient regional perfusion changes without true ischemia. Together, these mechanisms offer a plausible explanation for how a global metabolic disorder can occasionally present with focal, symmetrical deep‐gray‐matter lesions and stroke‐like deficits. The clinical presentation and MRI findings were key to prompting metabolic evaluation. The combination of acute hemiparesis, history of developmental delay and hypotonia, and bilateral globus pallidus signal abnormalities without diffusion restriction strongly suggested a metabolic rather than vascular etiology. Elevated urinary GHB and confirmatory ALDH5A1 sequencing established the diagnosis. Neuroimaging in SSADH deficiency typically shows symmetrical T2 hyperintensities of the globus pallidus, subthalamic nuclei, and dentate nuclei [4, 8, 10, 11, 12, 14, 17]. Additional features may include delayed myelination, cerebellar atrophy, or brainstem thinning. These patterns overlap with other neurometabolic disorders, such as GA1, L‐2‐hydroxyglutaric aciduria, and mitochondrial encephalopathies, though SSADH deficiency usually lacks the diffuse white‐matter changes seen in many of these conditions. Spectroscopy may demonstrate increased glutamine/glutamate peaks. Toxin‐related conditions such as vigabatrin exposure and kernicterus can also involve the globus pallidus, but isolated, symmetrical deep‐gray involvement should prompt consideration of SSADH deficiency.

Clinically, SSADH deficiency manifests with variable neurodevelopmental symptoms, including developmental delay, hypotonia, ataxia, behavioral disturbances, and seizures in approximately half of cases [4, 15, 18]. EEG findings are nonspecific and may show background slowing, focal or generalized epileptiform discharges, or be normal [14].

Over 120 pathogenic ALDH5A1 variants have been reported, with no known genotype–phenotype correlation; siblings carrying identical variants may show widely differing clinical severity [2, 19]. This case's atypical presentation underscores the need for ongoing reporting of the natural history of SSADH and international registry efforts.

Management of SSADH deficiency remains supportive. Treatment focuses on seizures, behavioral disturbances, and sleep disorders, though responses are variable. Vigabatrin has been trialled to reduce GHB accumulation by limiting upstream SSA production; however, mixed clinical responses and concerns regarding retinal toxicity restrict its use [13, 20]. Other supportive strategies include physical, occupational, and speech therapies, tailored to address specific motor and cognitive impairments [1].

Emerging therapies are aimed at addressing the underlying molecular defect. Experimental approaches include gene therapy to restore ALDH5A1 function, enzyme replacement strategies, pharmacological chaperones, and antioxidants such as N‐acetylcysteine to reduce oxidative stress [7, 15]. Stop‐codon readthrough therapies are also under investigation for nonsense variants. Early diagnosis may enable participation in future clinical trials, underscoring the importance of considering SSADH deficiency in atypical presentations [7].

This case highlights the importance of evaluating IEM in infants with acute neurological deficits when standard stroke evaluations are unrevealing. Although metabolic stroke in SSADH deficiency is rare, it may be under‐recognized, particularly during febrile illness or metabolic stress. Timely recognition supports appropriate counseling, management, and potential access to emerging therapies. Further research is needed to characterize mechanisms behind stroke‐like episodes in SSADH deficiency and determine whether they represent a distinct clinical subset or part of a broader disease spectrum.

## 4 Conclusions

In conclusion, this case broadens the recognized spectrum of SSADH deficiency by demonstrating that metabolic stroke, though rare, can occur in early infancy. While the disorder is usually characterized by chronic neurodevelopmental impairment, acute focal neurological events should prompt consideration of a metabolic cause. Given the risk of under‐recognition, metabolic evaluation with urinary organic acids and confirmatory genetic testing is essential. As no definitive cure exists, early diagnosis supports appropriate management, counseling, and access to emerging therapies. Further clarification of the mechanisms underlying stroke‐like episodes may guide future treatment development and improve outcomes.

## Author Contributions

S.K., C.D., R.J.L., E.M.‐L., O.‐P.Q.: directly involved in the patient's care. S.K. and C.D.: planning of manuscript. S.K. and C.D.: drafting of manuscript. S.K., C.D., R.J.L., E.M.‐L., O.‐P.Q., F.N., M.C., J.Y.‐L.: revision of the manuscript. All authors approve the version to be published and agree to be accountable for all aspects of the work in ensuring that questions related to the accuracy or integrity of any part of the work are appropriately investigated and resolved.

## Funding

The authors have nothing to report.

## Ethics Statement

All procedures followed were in accordance with the ethical standards of the responsible committee on human experimentation (institutional and national) and with the Declaration of Helsinki, 1975, as revised in 2000. This article does not contain any studies with animal subjects performed by any of the authors.

## Consent

Informed and written consent was obtained from the caregiver for being included in the article.

## Conflicts of Interest

The authors declare no conflicts of interest.

## Data Availability

The data that support the findings of this study are available from the corresponding author upon reasonable request.

## References

[1] P. Malaspina , “Succinic Semialdehyde Dehydrogenase Deficiency (SSADHD): Pathophysiological Complexity and Multifactorial Trait Associations in a Rare Monogenic Disorder of GABA Metabolism,” Neurochemistry International 99 (2016): 72–84. 27311541 10.1016/j.neuint.2016.06.009 PMC5028283

[2] N. A. Julia‐Palacios , O. Kuseyri Hübschmann , M. Olivella , et al., “The Continuously Evolving Phenotype of Succinic Semialdehyde Dehydrogenase Deficiency,” Journal of Inherited Metabolic Disease 47, no. 3 (2024): 447–462. 38499966 10.1002/jimd.12723

[3] K. E. Glinton , C. Gijavanekar , A. Rajagopal , et al., “Succinic Semialdehyde Dehydrogenase Deficiency: A Metabolic and Genomic Approach to Diagnosis,” Frontiers in Genetics 15 (2024): 1405468. 39011401 10.3389/fgene.2024.1405468 PMC11247174

[4] P. L. Pearl , K. M. Gibson , M. T. Acosta , et al., “Clinical Spectrum of Succinic Semialdehyde Dehydrogenase Deficiency,” Neurology 60, no. 9 (2003): 1413–1417. 12743223 10.1212/01.wnl.0000059549.70717.80

[5] P. Wang , F. Cai , L. Cao , et al., “Clinical Diagnosis and Mutation Analysis of Four Chinese Families With Succinic Semialdehyde Dehydrogenase Deficiency,” BMC Medical Genetics 20, no. 1 (2019): 88. 31117962 10.1186/s12881-019-0821-z PMC6532217

[6] I. Tokatly Latzer , J. B. Roullet , W. Afshar‐Saber , et al., “Clinical and Molecular Outcomes From the 5‐Year Natural History Study of SSADH Deficiency, a Model Metabolic Neurodevelopmental Disorder,” Journal of Neurodevelopmental Disorders 16, no. 1 (2024): 21. 38658850 10.1186/s11689-024-09538-9 PMC11044349

[7] M. Didiasova , A. Banning , H. Brennenstuhl , et al., “Succinic Semialdehyde Dehydrogenase Deficiency: An Update,” Cells 9, no. 2 (2020): 19. 10.3390/cells9020477 PMC7072817 32093054

[8] O. Afacan , E. Yang , A. P. Lin , et al., “Magnetic Resonance Imaging (MRI) and Spectroscopy in Succinic Semialdehyde Dehydrogenase Deficiency,” Journal of Child Neurology 36, no. 13–14 (2021): 1162–1168. 33557675 10.1177/0883073821991295 PMC8349937

[9] P. L. Pearl , E. J. Novotny , M. T. Acosta , C. Jakobs , and K. M. Gibson , “Succinic Semialdehyde Dehydrogenase Deficiency in Children and Adults,” Annals of Neurology 54, no. Suppl 6 (2003): S73–S80. 12891657 10.1002/ana.10629

[10] M. T. Acosta , J. Munasinghe , P. L. Pearl , et al., “Cerebellar Atrophy in Human and Murine Succinic Semialdehyde Dehydrogenase Deficiency,” Journal of Child Neurology 25, no. 12 (2010): 1457–1461. 20445195 10.1177/0883073810368137 PMC3155424

[11] K. Y. Wang , P. B. Barker , and D. D. Lin , “A Case of Acute Onset Succinic Semialdehyde Dehydrogenase Deficiency: Neuroimaging Findings and Literature Review,” Child's Nervous System 32, no. 7 (2016): 1305–1309. 10.1007/s00381-015-2942-9 26499347

[12] K. J. Kim , P. L. Pearl , K. Jensen , et al., “Succinic Semialdehyde Dehydrogenase: Biochemical‐Molecular‐Clinical Disease Mechanisms, Redox Regulation, and Functional Significance,” Antioxidants & Redox Signaling 15, no. 3 (2011): 691–718. 20973619 10.1089/ars.2010.3470 PMC3125545

[13] S. Yoganathan , G. Arunachal , L. Kratz , et al., “Metabolic Stroke: A Novel Presentation in a Child With Succinic Semialdehyde Dehydrogenase Deficiency,” Annals of Indian Academy of Neurology 23, no. 1 (2020): 113–117. 32055132 10.4103/aian.AIAN_213_18 PMC7001443

[14] P. L. Pearl , M. DiBacco , C. Papadelis , et al., “Succinic Semialdehyde Dehydrogenase Deficiency: Review of the Natural History Study,” Journal of Child Neurology 36, no. 13–14 (2021): 1153–1161. 33393837 10.1177/0883073820981262 PMC8254814

[15] H. Dong , X. Ma , Z. Chen , et al., “Clinical Features and ALDH5A1 Gene Findings in 13 Chinese Cases With Succinic Semialdehyde Dehydrogenase Deficiency,” BMC Medical Genomics [Electronic Resource] 17, no. 1 (2024): 158. 38862963 10.1186/s12920-024-01925-4 PMC11165735

[16] I. Tokatly Latzer , M. Bertoldi , N. Blau , et al., “Consensus Guidelines for the Diagnosis and Management of Succinic Semialdehyde Dehydrogenase Deficiency,” Molecular Genetics and Metabolism 142, no. 1 (2024): 108363. 38452608 10.1016/j.ymgme.2024.108363 PMC11073920

[17] C. Sergi and B. Parayil Sankaran , Succinic Semialdehyde Dehydrogenase Deficiency (StatPearls Publishing, 2024). 32809559

[18] P. Stenson , “Human Gene Mutation Database (HGMD): 2003 Update,” Human Mutation 21, no. 6 (2003): 577–581. 12754702 10.1002/humu.10212

[19] M. Parezanovic , N. Ilić , S. Ostojić , et al., “Sensorineural Hearing Loss in a Child With Succinic Semialdehyde Dehydrogenase Deficiency,” Balkan Journal of Medical Genetics 26, no. 1 (2023): 63–68. 37576789 10.2478/bjmg-2023-0008 PMC10413887

[20] A. Gropman , “Vigabatrin and Newer Interventions in Succinic Semialdehyde Dehydrogenase Deficiency,” Annals of Neurology 54, no. Suppl 6 (2003): S66–S72. 12891656 10.1002/ana.10626

